# Isolated splenic tuberculosis mimicking a solid splenic neoplasm in an immunocompetent patient: A surgical case report

**DOI:** 10.1016/j.ijscr.2025.111917

**Published:** 2025-09-09

**Authors:** Joyce Chege, Daniel.O. Otieno, Nicholas Nyamai, Lucy W. Muchiri, Andrew Ndonga

**Affiliations:** a Department of Surgery, College of Surgeons of East, Central, Southern Africa (COSECSA), The Mater Misericordiae Hospital, Nairobi, Kenya; b Department of Surgery, University of Nairobi (UoN), Kenyatta National Hospital, Nairobi, Kenya; c Department of Surgery, College of Pathologists of East, Central, Southern Africa (COPECSA), The Mater Misericordiae Hospital, Nairobi, Kenya

**Keywords:** Case report, Splenectomy, Immunocompetent, Splenic neoplasm, Splenic tuberculosis

## Abstract

**Introduction and importance:**

Tuberculosis is the leading cause of death by an infectious agent globally, especially among immunocompromised patients. Extrapulmonary tuberculosis (EPTB) can occur as a sequela of pulmonary tuberculosis through lympho-hematogenous or miliary spread, or can occur in isolation. EPTB rarely occurs in isolation in a single organ, especially in immunocompetent patients. Limited data exist on the incidence of isolated splenic tuberculosis in immunocompetent patients.

**Case presentation:**

This report highlights a case of a 45-year-old immunocompetent female with a one-month history of dull left upper quadrant pain. A contrast-enhanced abdominal CT scan revealed a solitary hypodense, heterogeneous, solid splenic mass occupying most of the splenic parenchyma, with an initial preoperative diagnosis of a splenic hemangioma. A near-total splenectomy was done, with histopathology revealing chronic granulomatous infection, suggesting tuberculous infection. In our scenario, the patient had a favourable outcome, receiving a six-month course of anti-tuberculous therapy and no surgical complications postoperatively.

**Clinical discussion:**

Splenic tuberculosis, although rare, can be classified as either micro-nodular, macro-nodular, miliary, or mixed types. The other subtypes commonly occur. However, macro-nodular splenic tuberculosis is rare and mimics benign and malignant splenic neoplasms radiologically, providing diagnostic challenges.

**Conclusion:**

Isolated splenic tuberculosis should be considered in the differential diagnosis of solitary splenic lesions, even in immunocompetent patients in TB-endemic areas. While timely diagnosis and anti-tubercular therapy may preserve splenic tissue in selected cases, surgery remains inevitable when diagnosis is uncertain, complications arise, or medical therapy fails. The case further highlights the importance of multidisciplinary team management.

## Highlights

• The rare incidence of having extrapulmonary tuberculosis affecting the abdominal viscera, especially the spleen.
• Occurrence of extrapulmonary tuberculosis in an immunocompetent patient with no previous history of pulmonary tuberculosis
• The role of a multidisciplinary team approach in the treatment and management of splenic lesions

## 1 Introduction

Globally, tuberculosis (TB) is the leading cause of death by an infectious agent, especially among immunocompromised patients [1]. According to the 2021 WHO global incidence report, the incidence of new infections is 134 per 100,000 population, with 23 % of these cases occurring in sub-Saharan Africa [2]. Extrapulmonary tuberculosis (EPTB) is a common sequela of the disease with dissemination to other sites via lympho-hematogenous or miliary spread in the background of HIV co-infection [3]. Abdominal TB occurs concomitantly with pulmonary tuberculosis in 10–15 % of cases, with high morbidity and mortality, as it presents with non-specific symptoms and signs [4]; hence, there is a delay in diagnosis and treatment.

Abdominal tuberculosis can occur in various forms: peritoneal, mesenteric lymphadenopathy, gastrointestinal, or visceral (among solid organs) [4]. Visceral tuberculosis is most commonly found in the genitourinary system, with 15 % of abdominal TB occurring in the non-genitourinary system [5]. The spleen is rarely involved and occurs with disseminated spread in immunocompromised patients [6].

Literature on isolated splenic tuberculosis in immunocompetent individuals is scarce, with most reports limited to single cases or small series [6]. The diagnosis is often obtained incidentally or in hindsight from histological specimens after splenectomy for other indications, such as trauma or solid neoplasms [6]. These cases present with non-specific abdominal symptoms and unremarkable laboratory findings, with radiologic features that mimic solid splenic neoplastic lesions, which contribute to diagnostic delays [7].

In TB-endemic regions, it should be considered in the differential diagnosis for unexplained solid splenic lesions once more common causes are excluded [8]. A high index of suspicion, coupled with timely image-guided aspiration or biopsy, may allow organ preservation. While anti-tubercular therapy is the cornerstone of treatment, surgery (splenectomy) is reserved for cases with diagnostic uncertainty, complications, or lack of response to medical therapy [9]. Splenectomy is associated with an increased lifelong risk of overwhelming post-splenectomy infection (OPSI), necessitating perioperative vaccination and patient education to reduce morbidity and mortality [10].

This report describes a case of a 45-year-old immunocompetent female with a solitary solid macro-nodular splenic lesion initially presumed to be a hemangioma based on radiologic features and later diagnosed as isolated splenic TB on histopathology. The patient was treated at a private tertiary faith-based academic institution in Nairobi, Kenya. Management involved an open near-total splenectomy followed by anti-tubercular therapy.

This case highlights the diagnostic challenges of this rare entity and underscores the value of a multidisciplinary team (MDT) approach. Furthermore, it is reported per the SCARE checklist 2025 and without the use of artificial intelligence (AI) tools [11].

## 2 Case presentation

We present a 45-year-old black African female residing in a formal urban residential area. She presented walking to our surgical outpatient clinic (SOPC) after a referral from the accident and emergency department with a one-month history of persistent dull left upper quadrant abdominal pain with no constitutional symptoms: fever, weight loss, night sweats, or a chronic cough. No previous history of comorbidities such as diabetes, human immunodeficiency virus (HIV) or a history of tuberculosis.

**Fig. 1:**
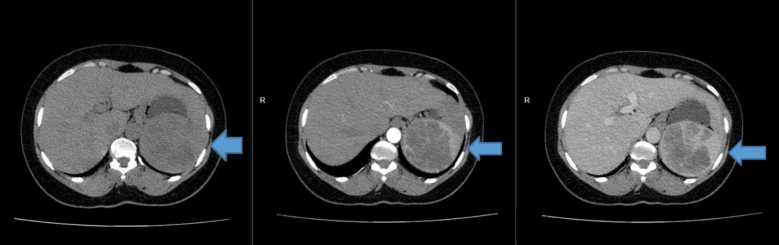
Contrast-enhanced axial CT scan images of the abdomen with the arrow showing a heterogeneous hypodense ill-defined vascular lesion within the splenic parenchyma.

**Fig. 2:**
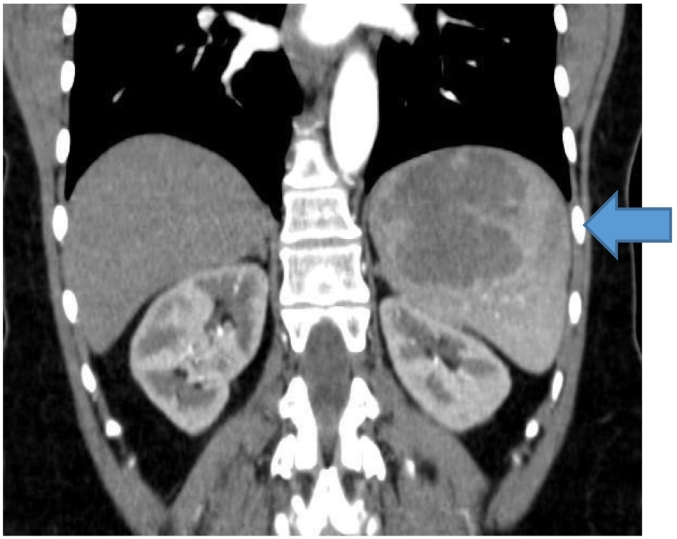
Coronal CT abdomen scan with the arrow demonstrating the mass in the superior pole of the spleen.

Her vital signs were within normal range. She did not have any lymphadenopathy on examination, and her abdominal examination revealed left-sided abdominal fullness with no associated tenderness. Laboratory investigations were done: ESR, complete blood count, liver and renal function tests, HIV, malaria testing, and peripheral smear, were unremarkable, with no evidence of hematologic malignancy or other common infectious causes of splenomegaly. Her fasting and random blood sugars were normal. Contrast-enhanced CT scan images of the abdomen revealed a heterogeneous, hypodense, ill-defined vascular lesion within the majority of the splenic parenchyma. The lesion was characterised by an irregular, lobulated outline measuring 9.2 × 8.2 × 6.9 cm, with a sleeve of normal splenic tissue inferiorly (Fig. 1, Fig. 2). There was no abdominal lymphadenopathy, ileocecal thickening, or ascites. A diagnosis of splenic hemangioma was made based on radiological features.

The patient was managed using a multidisciplinary team (MDT) approach involving the general surgeon, radiologist and pathologist teams. A decision to perform an open splenectomy was made due to the size of the hemangioma, ease of haemorrhage control through open vs. laparoscopic surgery, and ease of leaving remnant splenic tissue. Furthermore, the patient was educated preoperatively on the risks associated with splenectomy, including overwhelming post-splenectomy infection (OPSI), and received vaccinations against *Streptococcus pneumoniae*, *Haemophilus influenzae* type b, and *Neisseria meningitidis* according to the institution's protocol two weeks before surgery.

Notably, given the preoperative confidence in the diagnosis of splenic hemangioma based on radiological findings, preoperative splenic embolisation was not pursued due to the patient's preference and the absence of any immediate risk of rupture as discussed by the MDT.

**Fig. 3:**
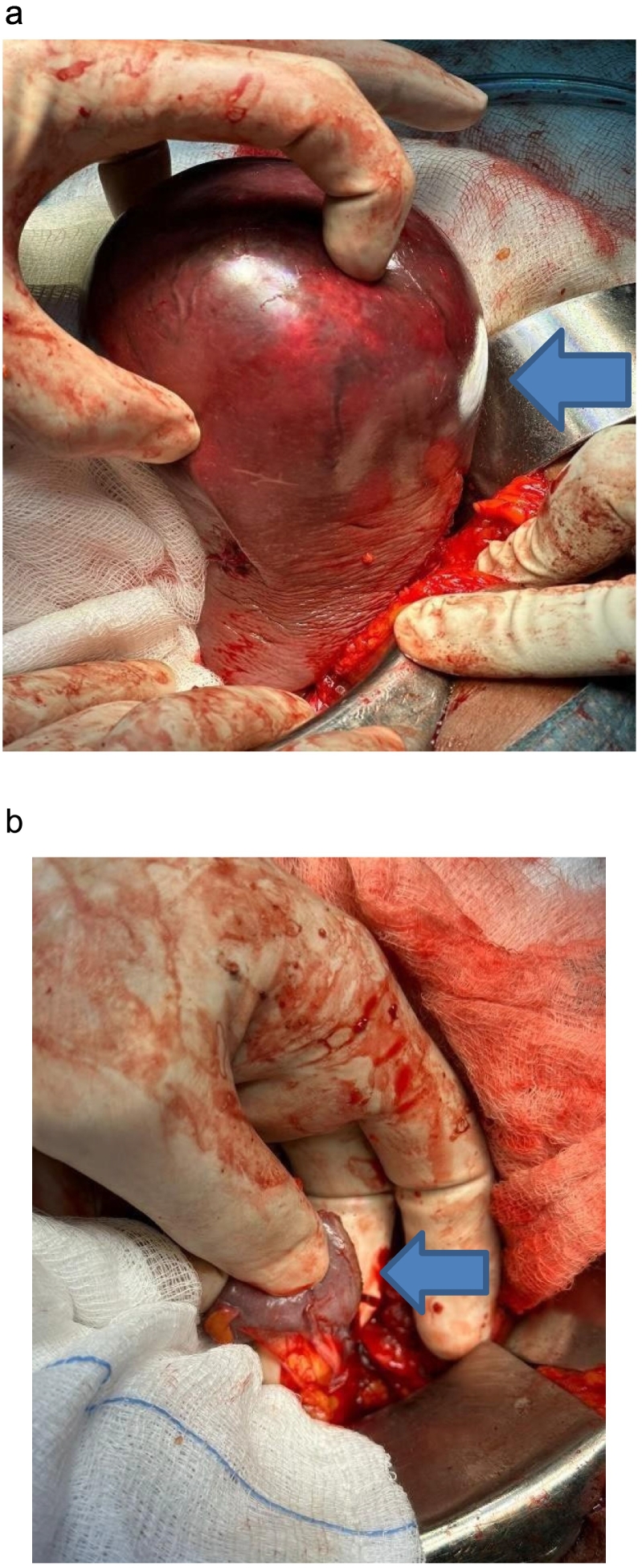
a. Intraoperative image, arrow demonstrating the mass occupying 90 % of the spleen. b. Remnant splenic tissue after near-total irregular splenectomy.

After detailed counselling regarding her operative risks, written informed consent was obtained from the patient. The general surgical team proceeded to perform an open, near-total splenectomy under general anaesthesia with the patient in the supine position. The approach was via an extended upper midline incision. Intraoperatively, no ascites nor abdominal lymphadenopathy was noted. Multiple adhesions were noted between the liver, the inferior diaphragmatic surface, the stomach, and the spleen. The splenic adhesions were freed bluntly and with a Ligasure energy device. The spleen was retracted medially and freed from its ligamentous attachments. The inferior vessels to the inferior pole of the spleen were spared, and the other splenic vessels were ligated, and a near-total splenectomy was done (Fig. 3a). The remaining splenic tissue was 4 cm in maximal length, inferiorly attached to a vascular pedicle (Fig. 3b). The excised spleen weighed 350 g grossly and measured 15 × 9 × 7 cm in size. The abdominal cavity was closed in layers with the rectus sheath closed with PDS suture 1, and the skin incision approximated with skin staples.

The nasogastric tube was placed after intubation to prevent acute postoperative gastric dilatation that may lead to haemorrhage and was removed after two days postoperatively. The patient was admitted to the general surgery ward for four days. During this period, she had normal vitals and blood cell counts reported with good pain control. She was discharged through the surgical clinic for follow-up. Furthermore, as per the OPSI protocol prevention strategies, the patient was educated on the warning signs to look out for incase of a fever, she was given antibiotic prophylaxis (oral amoxicillin 250 mg once daily for 12 months), vaccine card with revaccination dates and counselled on the importance of adherence to her medical therapy.

The primary surgeon who performed the procedure is a consultant general surgeon with extensive experience in open splenectomy and abdominal procedures in conjunction with the hospital's surgical team, who managed the patient during the perioperative period and follow-up in the surgical clinics.

**Fig. 4:**
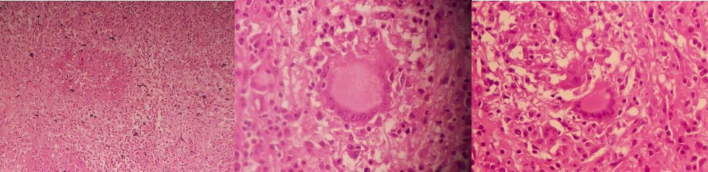
Histopathological examination showing epithelioid cell granulomas with Langhans-type multinucleated giant cells (granulomatous inflammation).

During her first follow-up visit to the clinic two weeks after discharge, her histopathology result revealed features of chronic granulomatous inflammation. The ZN stain was negative for Acid-fast bacilli (AFB), and the QuantiFERON TB Gold test was positive for tuberculosis (Fig. 4), no GeneXpert MTB/RIF testing or cultures were performed in this case, as the tissue was fixed in formalin for histopathological analysis based on the initial impression of splenic hemangioma.

**Fig. 5:**
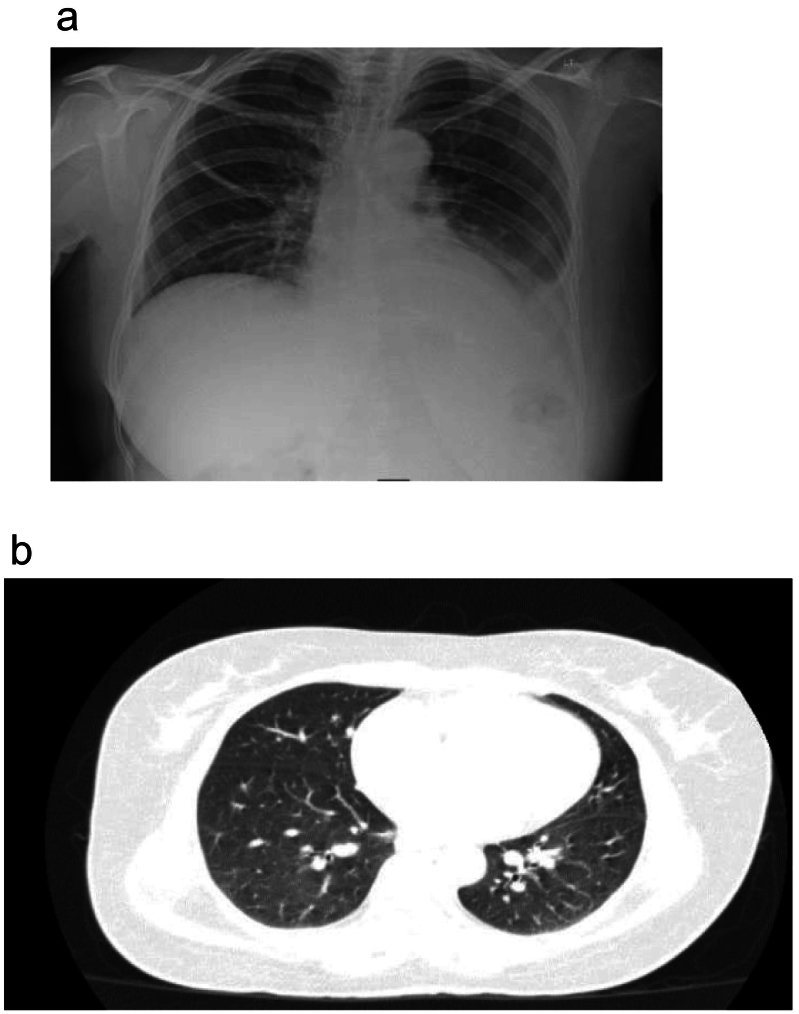
a: Chest X-ray, no features of pulmonary tuberculosis. b: Chest CT scan with no features suggestive of pulmonary tuberculosis.

**Fig. 6:**
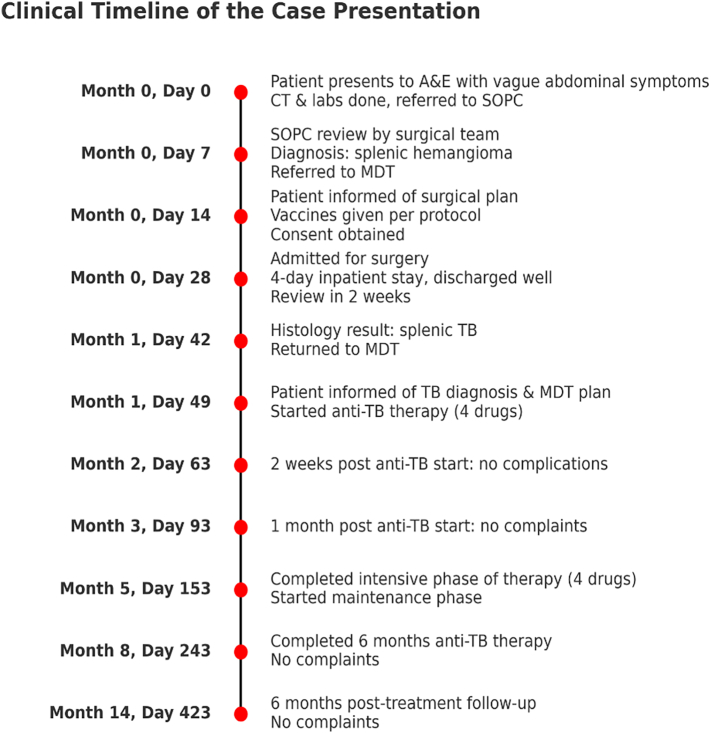
Timeline summarising the clinical course of the patient from initial presentation to diagnosis of splenic tuberculosis, treatment, and follow-up.

The patient was further discussed with the histopathological findings in the MDT, and a recommendation to perform a chest X-ray and pulmonary CT scan was made to rule out pulmonary tuberculosis. No features of pulmonary tuberculosis (Fig. 5a and b) or extrapulmonary tuberculosis were detected. In view of the increasing size of her spleen preoperatively, suggesting active extrapulmonary disease, the patient was commenced on a six-month course of anti-TB therapy of: Isoniazid, rifampicin, pyrazinamide, and ethambutol. She completed her course of treatment with good regeneration of the remnant spleen and without any complications on follow-up. She has been up-to-date with her revaccinations. She is still on follow-up in the SOPC. (Fig. 6)

## 3 Clinical discussion

Patients with isolated splenic TB can present with a variety of nonspecific symptoms, depending on the subtype of splenic tuberculosis, which includes micro-nodular, macro-nodular, miliary, or mixed types. The clinical symptoms range from fever to vague abdominal pain and splenomegaly [8]. Laboratory investigations for EPTB are usually not pathognomic; they may reveal elevated inflammatory markers, such as ESR and subtle cytopenia, which can lead to a workup focused more on malignancy than infection, especially in an immunocompetent patient [6]. In our scenario, the patient presented with nonspecific, vague abdominal pain with normal laboratory findings. Therefore, highlighting the importance of radiologic imaging in identifying the lesion as seen in other case reports [12].

Radiologically, differentiating between solitary solid splenic masses is challenging due to their overlapping radiologic features [7]. Splenic TB can occur in four subtypes, with the macro-nodular solitary subtype closely mimicking benign or malignant splenic neoplasms. Early in its course, macronodular solitary splenic TB appears hypodense on a CT scan with a heterogeneous outline and central necrosis. As the disease progresses, the outline of the lesion may calcify, accompanied by abdominal lymphadenopathy, ileocecal thickening, and ascites (9) depending on the disease stage and individual patient factors.

In contrast, splenic hemangioma on CT scan may appear as a solitary homogenous mass. Following contrast administration, they usually show peripheral ring enhancement in the arterial phase with progressive centripetal fill-in the venous and delayed phase. However, this enhancement pattern can be variable, with some lesions showing homogeneous enhancement or even lack of enhancement in the centre on delayed images [7]. The diagnostic challenge arises because both splenic TB and hemangioma may have calcific ring enhancement on CT, as was seen in our scenario. Differentiation may be aided by evaluating patterns on triple-phase CT scans correlating with clinical history and assessing for associated findings such as abdominal lymphadenopathy and the patient's immune status [13]. The definitive diagnosis, therefore, falls on histological confirmation via a percutaneous biopsy or diagnostic surgical intervention [14].

Although image-guided percutaneous biopsy is widely used for diagnosing many splenic lesions and is generally considered safe [15], its application is limited when vascular lesions such as hemangiomas are suspected. This is because biopsies in these cases carry a substantial risk of haemorrhage and splenic rupture, which can lead to serious complications. In our patient, the radiological appearance suggested a large vascular mass consistent with a hemangioma, making biopsy a high-risk procedure. Therefore, a diagnostic biopsy was deferred to avoid these risks. Instead, an open near-total splenectomy was performed, which served both diagnostic and therapeutic purposes. This approach allowed for safe excision of the lesion, minimised the risk of intraoperative bleeding, and facilitated preservation of some splenic tissue to reduce the likelihood of overwhelming post-splenectomy infection (OPSI) [10]. Thus, the decision to proceed directly to splenectomy was primarily guided by the safety concerns associated with biopsy of vascular lesions and the need for definitive management.

However, if the lesion wasn't vascular, the diagnosis of splenic TB may have been made histologically via a percutaneous image-guided core biopsy, as shown in other case reports [16].

Following diagnosis of splenic TB, evidence-based guidelines suggest that the management of EPTB with anti-TB therapy would be for six months for active disease [17]. Some studies have advocated for medical management alone in immunocompetent patients without systemic symptoms, rupture, or abscess formation, primarily in miliary type or calcified splenic TB, where short-term anti-TB chemotherapy is given, followed by clinical observation for resolution of symptoms. If medical therapy fails, surgery is warranted [9]. This applies with necrotic TB lesions, where medical therapy can still be effective depending on the extent of disease and clinical response and surgical management is typically reserved for cases unresponsive to medical treatment or with complications [6].

Furthermore, clinicians should be wary that some patients may paradoxically present with worsening symptoms after initiation of anti-TB medications, with increased inflammatory markers, and the appearance of new lesions, both clinical and radiological [16]. This patient was successfully managed on a six-month course of anti-TB therapy with complete clinical response and preservation of healthy splenic tissue. Patient education on the importance of completing antibiotic prophylaxis doses, and vigilance for the symptoms of infection is also important following a splenectomy [10].

## 4 Conclusion

Isolated splenic tuberculosis is a rare form of EPTB, particularly in immunocompetent individuals with no prior history of pulmonary tuberculosis. The pathology mimics solid splenic neoplasms on radiology and should be considered as a differential diagnosis in TB-endemic regions. Careful multidisciplinary evaluation and appropriate use of surgery and anti-tuberculous therapy can lead to favourable outcomes, preserving splenic tissue and minimising complications such as overwhelming post-splenectomy infection (OPSI).

## 5 Learning points

1. Isolated splenic TB can present with nonspecific symptoms and imaging findings mimicking splenic neoplasms, requiring high clinical suspicion, especially in TB-endemic regions and among immunocompetent patients.
2. The multidisciplinary team (MDT) approach is essential for timely diagnosis and appropriate treatment, balancing surgery and medical therapy.
3. Anti-TB therapy remains the cornerstone of treatment for splenic TB, with surgery reserved for select cases.

## 6 Limitations of the study

1. This is a single-patient case report, and the findings cannot be generalised. However, it highlights an unusual presentation and management approach that may inform clinicians when encountering similar cases.
2. Potential bias: The successful outcomes of the presented case could introduce bias, since there was no comparison with a similar case managed non-surgically.

## Author contribution

The corresponding author, Dr Joyce Chege, led the conceptualisation and writing of the first draft. Dr Dan Otieno, Dr Nicholas Nyamai, Prof. Lucy Muchiri and Dr Andrew Ndonga all contributed equally to the review and editing of the original draft.

## Consent

Written informed consent was obtained from the patient for publication and the accompanying images. A copy of the written consent is available for review by the Editor-in-Chief of this journal on request.

## Ethical approval

No ethical approval is needed for a case report in our institution, The Mater Misericordiae Hospital, Nairobi, Kenya. The institution is a training centre under the College of Surgeons of East, Central and Southern Africa (COSECSA) program.

## Guarantor

The corresponding author.

## Research registration number

1. Name of the registry: N/A.
2. Unique Identifying number or registration ID: N/A.
3. Hyperlink to your specific registration (must be publicly accessible and will be checked): N/A.

## Funding

None.

## Conflict of interest statement

N/A.
